# Extracellular matrix re-normalization to improve cold tumor penetration by oncolytic viruses

**DOI:** 10.3389/fimmu.2024.1535647

**Published:** 2025-01-08

**Authors:** Geofrey F. Soko, Benson K. Kosgei, Stephene S. Meena, Ying Jing Ng, Huihui Liang, Bing Zhang, Qingjun Liu, Tielong Xu, Xinju Hou, Ray P. S. Han

**Affiliations:** ^1^ Jiangzhong Cancer Research Center, Jiangxi University of Chinese Medicine, Nanchang, Jiangxi, China & Jiangxi Engineering Research Center for Translational Cancer Technology, Jiangxi University of Chinese Medicine, Nanchang, China; ^2^ Ocean Road Cancer Institute, Dar es Salaam, Tanzania; ^3^ The Affiliated Hospital of Jiangxi University of Chinese Medicine, Nanchang, China; ^4^ Biosensor National Special Laboratory & Key Laboratory for Biomedical Engineering of Education Ministry, Dept. of Biomedical Engineering, Zhejiang University, Hangzhou, China; ^5^ Evidence-based Medicine Research Center, Jiangxi University of Chinese Medicine, Nanchang, China; ^6^ Dept. of Rehabilitation, Nanchang Hongdu Hospital of Chinese Medicine, Nanchang, China

**Keywords:** cold tumors, tumor immune microenvironment, immunotherapy, oncolytic viruses, tumor-infiltrating immune cells, tumor ECM barriers

## Abstract

Immunologically inert or cold tumors pose a substantial challenge to the effectiveness of immunotherapy. The use of oncolytic viruses (OVs) to induce immunogenic cell death (ICD) in tumor cells is a well-established strategy for initiating the cancer immunity cycle (CIC). This process promotes the trafficking and infiltration of CD8+ T cells into tumors, thereby eliciting a tumor-specific immune response. Despite the potential of OVs for handling cold tumors, clinical outcomes have fallen short of expectations. To better understand the obstacles faced by oncolytic virus immunotherapy (OVI), we would like to revisit the OV issue. Growing evidence indicates that limited intratumoral penetration and inadequate intratumoral distribution of OVs are critical factors contributing to the suboptimal response to OVI. Aberrant expressions of matrix proteins by cancer-associated fibroblasts (CAFs) alter the mechanical properties of the tumor extracellular matrix (ECM). This results in increased ECM desmoplasia and elevated intratumoral interstitial fluid pressure (IFP), creating physical barriers that impede the penetration and dissemination of OVs within tumors. This review explores the latest advancements in strategies designed to improve the intratumoral penetration of OVs to facilitate the penetration of tumor-infiltrating lymphocytes (TILs) into cold tumors. Additionally, we investigated current clinical trials and challenges associated with translating these strategies into clinical practice to improve patient outcomes.

## 1 Introduction

Cancer immunotherapy has fundamentally transformed cancer care for a wide range of tumor types, shifting the treatment paradigm towards the hope of achieving long-term control of tumors that are naïve to conventional therapies such as surgery, chemotherapy, and radiotherapy (1, 2). The current landscape of immunotherapeutics encompasses immune checkpoint inhibitors (ICIs), adoptive cell therapies, oncolytic viruses (OVs), and cancer vaccines. Despite these advancements, the clinical benefits of immunotherapy have been observed in only a small subset of cancer patients. The efficacy of immunotherapy is strongly influenced by the presence of a high infiltration of lymphocytes and a reduced abundance of immunosuppressive cells (3). T cells are the primary anti-tumor effector lymphocytes and the focus of the majority of immunotherapeutic strategies. The density and distribution of T cell infiltration serve as a critical indicator of the response to immunotherapy interventions. For instance, the effectiveness of ICIs is better in hot tumors with a high lymphocyte penetration than in immunologically inert or cold tumors that lack infiltrating lymphocytes (4, 5).

The pattern of T cell infiltration within tumors is heavily shaped by the tumor extracellular matrix (ECM), which is notably denser and stiffer than a normal ECM. Immunologically cold tumors, which lack significant immune cell infiltration, are commonly characterized by this dense and stiff ECM, a condition known as desmoplasia (6), which is a result of tumor-induced ECM remodeling (7). The altered expression of matrix proteins, including collagens, laminins, and fibronectin, as well as glycans such as glycosaminoglycans and proteoglycans, changes the mechanical properties of the tumor ECM, leading to increased ECM density, stiffness, and interstitial fluid pressure (IFP) (8). This aberrant tumor ECM creates physical barriers that hinder the penetration of immune cells, drugs, and chimeric antigen receptor T (CAR-T) cells, thereby adversely affecting the efficacy of cancer treatment (9, 10).

**Figure 1:**
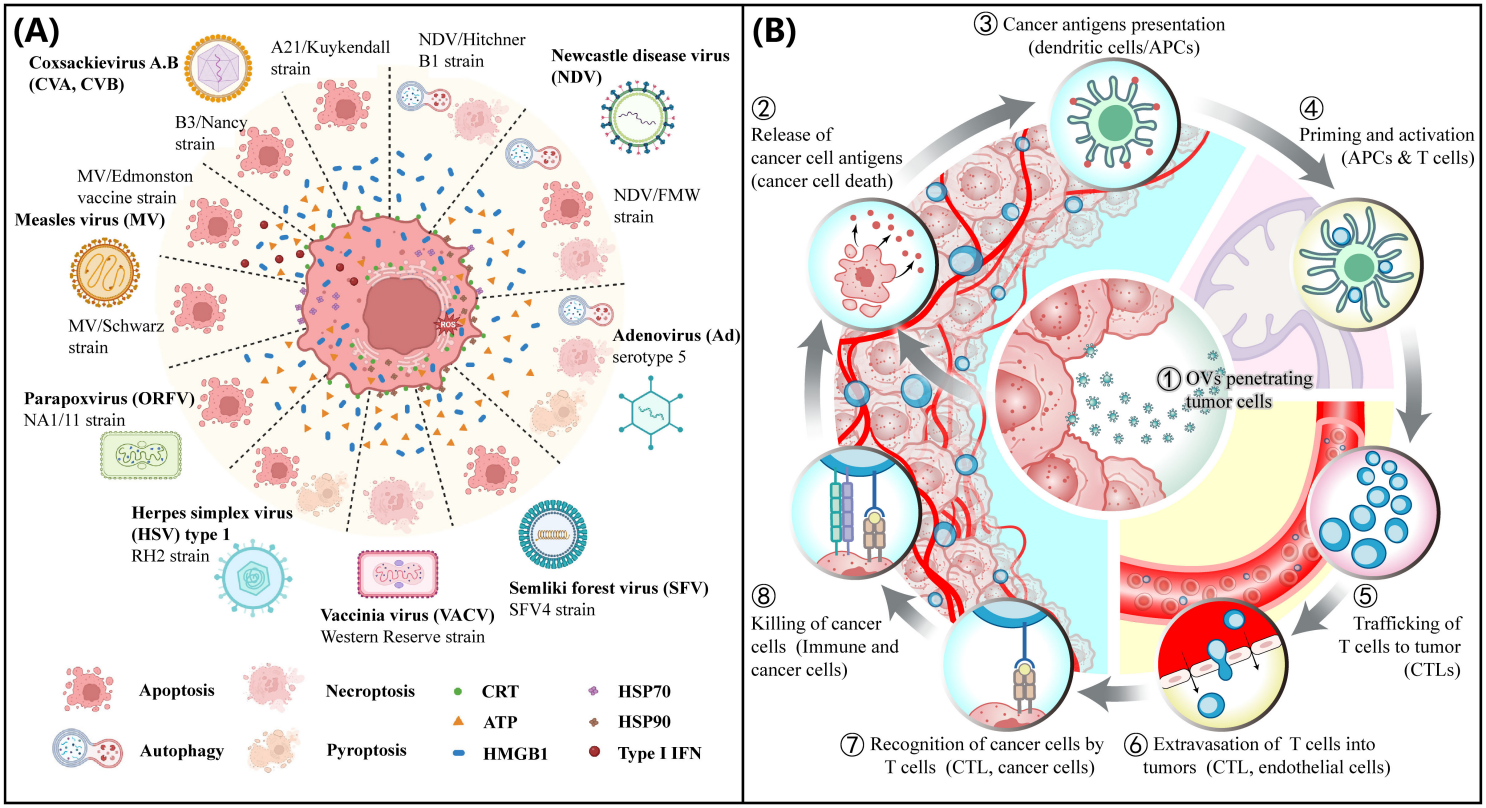
OV mediated tumor immunogenicity. **(A)** Immunogenic cell death (ICD). Immunogenic cell death pathways induced by OVs. Several natural OVs, including coxsackievirus A, B (CVA, CVB), Newcastle disease virus (NDV), adenovirus (Ad), semliki forest virus (SFV), measles virus (MV), herpes simplex virus (HSV) and poxviruses, such as vaccinia virus (VACV) and parapoxvirus (ORFV) can induce ICD. **(A)** and a slightly modified legend are reproduced under the terms of the Creative Commons Attribution License (CC BY 4.0) from Ref. (12). ^©^ 2023, Palanivelu, Liu and Lin. **(B)** Cancer immunity cycle (CIC). Cancer Immunity Cycle (CIC) is triggered when tumor cells are exposed to immunogenic cell death (ICD) inducers such as oncolytic viruses (OVs). As these cells become damaged or undergo ICD, they express damage-associated molecular patterns (DAMPs). The release of DAMPs, including high mobility group protein B1 (HMGB1) from the nucleus and the translocation and exposure of calreticulin (ecto-CRT) on the cell surface by stressed or dying tumor cells, enhances the presentation of tumor antigens, which are then captured by dendritic cells (DCs) at the tumor site. As DCs mature, they migrate to the lymph nodes, where they process tumor antigens and present them on Human Leukocyte Antigen class I (HLA-I) molecules to CD8+ T cells. This action primes and activates cytotoxic T lymphocytes (CTLs), preparing them for trafficking and infiltration into tumors to elicit a tumor-specific immune response.

Oncolytic virus immunotherapy (OVI) harnesses viruses to selectively infect and lyse tumor cells (11). The ensuing virus-mediated immunogenic cell death (ICD) (**Figure 1A**) releases tumor antigens (TAs) and damage-associated molecular patterns (DAMPs) that are captured by antigen-presenting dendritic cells (APCs) inside the tumor and presented to naïve T cells in the lymph nodes, priming them to attack tumor cells (12–17). The primed and activated CD4+ and CD8+ T cells are trafficked into the tumor to exert their effector functions, thus making immunologically cold tumors hot (18–21). The dying tumor cells release TAs and DAMPs, triggering activation and infiltration of T cells leading to an immune activation cycle referred to as the cancer immunity cycle (CIC) (**Figure 1B**). OVI-mediated conversion of cold tumors to hot provides an opportunity for the development of combination therapies that involve novel immunotherapeutic approaches such as immune checkpoint blockade (ICB), adoptive T-cell therapy, and cancer vaccines (22–27). Accumulating evidence implicates the limited intratumoral penetration and poor distribution of oncolytic viruses as two key factors to the poor response to OVI (28, 29). Aberrant expressions of matrix proteins (collagens, laminins, fibronectin, and elastin) and glycans (glycosaminoglycans (GAGs) and proteoglycans (PGs)) by cancer-associated fibroblasts (CAFs) alter the mechanical properties of the tumor ECM, leading to an increase in ECM desmoplasia and an elevated intratumoral IFP (8), creating physical barriers that impede the penetration and distribution of OVs. Overcoming these barriers is a crucial step in unlocking the full potential of OVI in modulating the tumor immune microenvironment (TIME). This review discusses the latest advancements in strategies to enhance the intratumoral penetration of OVs, thereby facilitating the infiltration of tumor-infiltrating lymphocytes (TILs) into cold tumors via the CIC process. We also explore clinical trials and challenges associated with translating these strategies into clinical practice to improve patient outcomes.

## 2 Barriers imposed by the tumor ECM to oncolytic virus penetration

In solid tumors, the tumor parenchyma is usually surrounded by an aberrantly organized ECM made of overexpressed components that contain variable proportions of fibrous proteins (collagen, fibronectin, laminin, and elastin), as well as, GAGs (hyaluronic acid (HA)), and PGs (chondroitin sulfate and heparan sulfate) (8, 30). Elevated collagen synthesis, fibrillogenesis, and crosslinking in desmoplastic tumors such as those of pancreatic adenocarcinoma are associated with an increase in ECM density, alignment, and stiffness (31).

CAFs are activated stromal cells arising from diverse cell types (tissue-resident fibroblasts, mesenchymal stem cells, endothelial cells, pericytes, epithelial cells, and bone marrow-derived stem cells) that are recruited during the genesis of tumor stroma (32, 33). They are responsible for the secretion of aberrant components of the tumor ECM (34). CAFs are a heterogeneous group of cells with different secretory and functional characteristics and are operationally classified into three subtypes including myofibroblast (myCAF), inflammatory (iCAF), and antigen-presenting CAF (apCAF) (35). The phenotypic conversion of stromal cells to CAFs is a result of signals from both tumor cells and recruited normal cells. For instance, tumor cell-derived transforming growth factor-β (TGF-β) induces the conversion of normal gastric resident fibroblasts to CAFs (36). Similarly, tumor-derived exosomes (TDEs) induce phenotypic conversion of vascular endothelial cells to CAFs (37). The myCAFs are the most common CAF sub-type and their abundance correlates with the physical properties of the tumor ECM. High intratumoral collagen deposition by myCAFs is responsible for the increased tumor ECM density (38), while myCAFs-exerted active forces on tumor cells and tumor ECM orchestrate the increase in ECM stiffness (39–41).

The tumor microenvironment (TME) exerts several factors that augment collagen secretion, fibrillogenesis, and crosslinking to further increase ECM desmoplasia. For instance, the hypoxic TME triggers an increase in the expression of lysyl oxidase (LOX) enzymes resulting in increased collagen crosslinking and stiffness (42, 43). Likewise, several cytokines, chemokines, and growth factors that are abundant in the TME exert pro-fibrotic effects to increase the secretion of ECM components by CAFs, tumor cells, and tumor-associated macrophages (TAMs). For example, high levels of TGF-β increase collagen secretion and remodeling by myCAFs and induce the M2 polarization of TAMs (44). The M2-polarized TAMs further promote ECM desmoplasia through the production of reactive nitrogen species and inducible nitric oxide synthase which exerts profibrotic effects on collagen-secreting cells (45). In addition, several pro-inflammatory cytokines that are abundant in the TME such as IL-1β and TNF-α promote tumor ECM desmoplasia by promoting the activation and secretory functions of ECM-secreting cells such as stellate cells in pancreatic ductal adenocarcinoma (PDAC) (46, 47).

**Figure 2:**
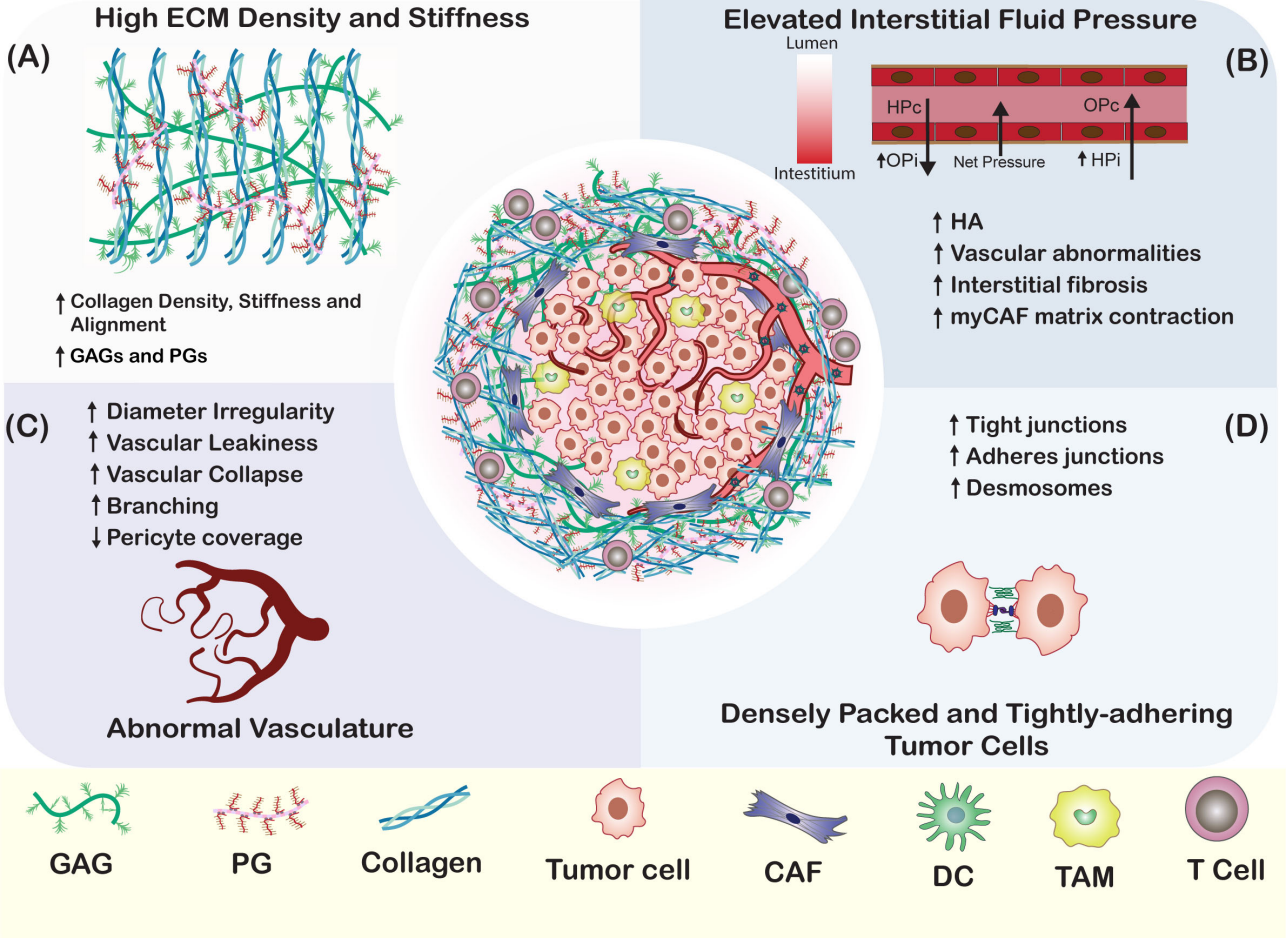
Physical Barriers to OV Penetration in Tumors. **(A)** In most solid tumors, the tumor parenchyma is encased by a dense ECM network composed of overexpressed proteins and glycans, such as collagen, fibronectin, laminin, HA, and PGs. This dense network hinders the penetration of OVs in a size-dependent manner (54). Factors contributing to the dense tumor ECM include the downregulation of matrix-degrading enzymes, the downregulation of collagen organizers, and the upregulation of lysyl oxidase. **(B)** Elevated interstitial fluid pressure results from an imbalance of forces that regulate fluid movement in and out of blood vessels. The tumor stroma is marked by increased interstitial oncotic pressure (OPi) and interstitial hydrostatic pressure (HPi), which create a net outward pressure that impedes the movement of materials towards the tumor interstitium (52). **(C)** Tumor stromal blood vessels are characterized by irregular diameters, increased permeability, excessive branching, and a lack of pericyte coverage (55). These factors are key contributors to the elevated stromal interstitial fluid pressure, which in turn impairs the movement of therapeutics to the tumor parenchyma. **(D)** Strong cell-cell adhesion between tumor cells, mediated by tight junctions, adherens junctions, and desmosomes, restricts the penetration of OVs towards the central regions of the tumor parenchyma (56).

In addition to collagen secretion, myCAFs secrete large amounts of HA, which contributes to altered physical properties of the tumor ECM (48, 49) and leads to an elevated intratumoral IFP due to the high hydrophilicity from its negative charge (50–52). Furthermore, the elevated HA in the tumor ECM is associated with the generation of abnormal tumor blood vessels with decreased patency and permeability (53). A combination of elevated IFP and hyperdense and stiffened ECM results in the collapse of intratumoral blood and lymphatic vessels (52). These features are associated with increased resistance to treatment by physically impeding the intratumoral penetration and distribution of drugs, therapeutic nanoparticles, OVs, antibodies, and immune cells (8–10) (**Figure 2**). Further barriers to penetration of the tumor parenchyma result from high tumor cell density within tumor nests and their tight adherence due to overexpression of cell-to-cell adhesion molecules (56, 57).

Substantial evidence suggests that excessive ECM desmoplasia is a significant factor that impairs the efficacy of OVI by hindering the intratumoral penetration of the viruses. Evidence from *in vitro* experiments of OV oncolysis has shown a decrease in oncolysis efficiency in three-dimensional (3D) spheroid culture conditions compared to two-dimensional (2D) monolayer culture conditions. For instance, oncolytic herpes simplex virus 1 (HSV-1) infection was highly effective in 2D cultured melanoma cell lines leading to a complete lysis of tumor cells while showing significant impairment in 3D cultures, which is consistent with ECM-mediated impairment of virus penetration and replication (28). Furthermore, the penetration of HSV-1 in human melanoma xenografts was found to be highly size-dependent. This was demonstrated by the similar impedance experienced by nanospheres loaded with quantum dots of comparable size to the virions (150 nm in diameter) and the superior penetration achieved by smaller dextran tracer molecules (20 nm in diameter) (54). Further, the degradation of ECM collagen using collagenase significantly enhanced the intratumoral penetration and distribution of HSV-1 in Mu89 melanoma xenografts (54). This treatment was associated with more robust and sustained tumor regression compared to the administration of HSV-1 alone. Additional evidence highlighting the role of tumor ECM barriers in hindering OV penetration was demonstrated in studies designed to enhance the susceptibility of tumor cells to OV infection. For instance, both rapamycin (Rap) and HA have been utilized to augment the susceptibility of gallbladder carcinoma (GBC) cells to infection by oncolytic myxoma virus (MYXV) (58, 59). However, the combination of MYXV with Rap (MYXV+Rap) significantly enhanced the susceptibility and lysis of GBC cell lines, but this effect was not observed in patient-derived xenografts (PDX) (58). Conversely, the combination of MYXV with HA (MYXV+HA) increased both the susceptibility and oncolytic efficacy in PDX models. This enhancement was attributed to hyaluronan-mediated induction of matrix metalloproteases 2 and 9 (MMP-2 and MMP-9) secretion, which facilitates the degradation of type-IV collagen (58, 60).

## 3 Overcoming tumor ECM barriers for oncolytic virus penetration

**Figure 3:**
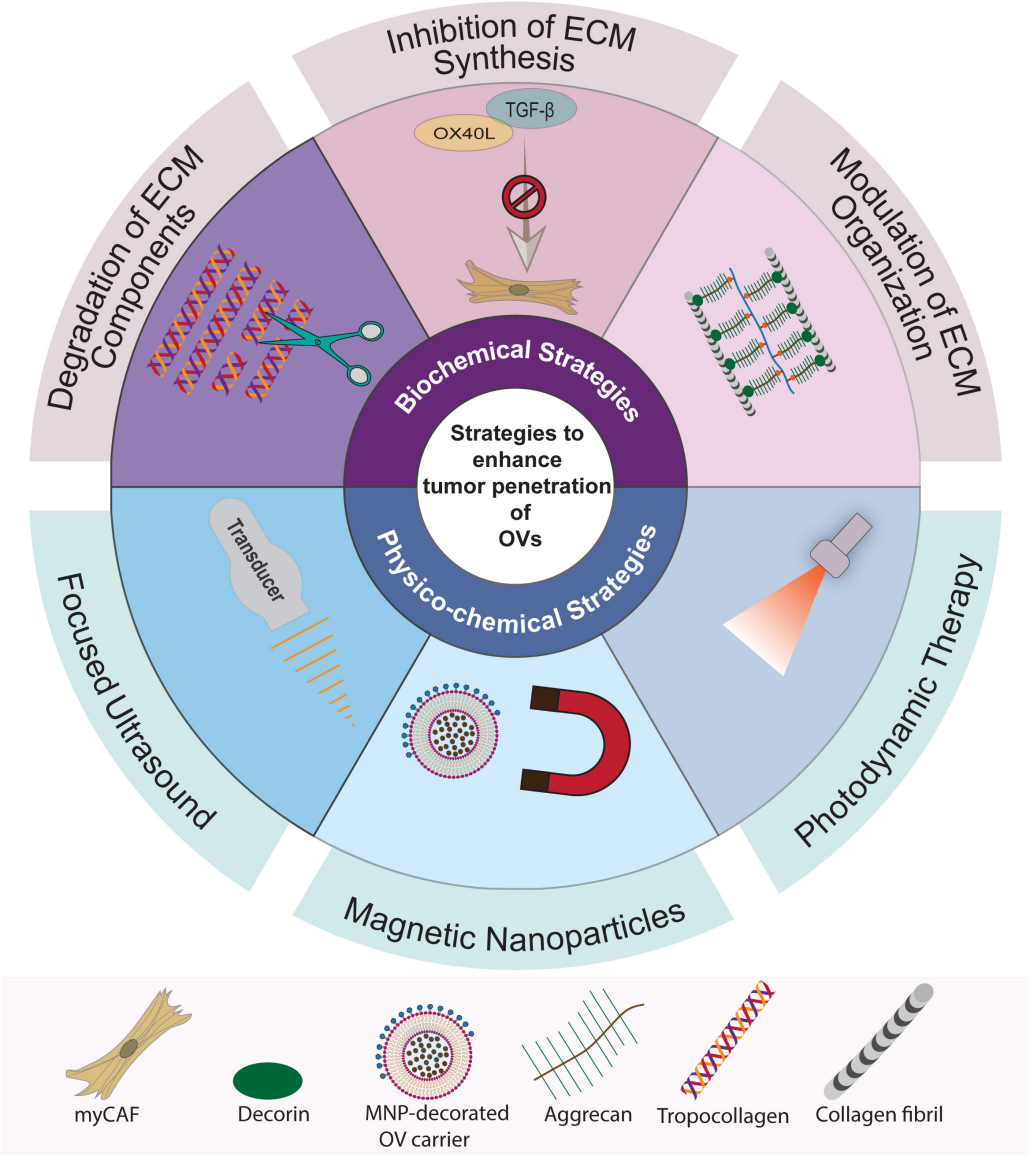
Summary of strategies to overcome ECM barriers for OV penetration in tumors. The strategies to enhance intratumoral penetration of OVs can be classified into two main categories: biochemical ECM modulation and physico-chemical disruption. Biochemical ECM modulation involves targeting specific pathways and macromolecules, including strategies for targeted degradation, inhibition of synthesis, and modulation of ECM organization. Physico-chemical disruption strategies, on the other hand, utilize sound, magnetic, and light energies to create mechanical or chemical effects in the tumor stroma to break down the ECM barriers. These methods encompass the use of focused ultrasound, magnetic nanoparticles (MNPs), and photodynamic therapy (PDT).

Recognition of the tumor ECM’s adverse role in obstructing intratumoral OV penetration has spurred research endeavors aimed at overcoming these barriers to improve the effectiveness of OVI. Advances in our understanding of the structural elements and molecular pathways that regulate tumor desmoplasia have paved the way for the development of strategies targeting specific components of the ECM. These ECM-targeting strategies can be categorically divided into two main types: biochemical modulation and physico-chemical disruption (**Figure 3**).

### 3.1 Biochemical strategies

Biochemical strategies for ECM modulation are designed to target specific components of the ECM that affect its physical characteristics. These approaches encompass the degradation of ECM constituents such as collagen, HA, and PGs; the inhibition of collagen synthesis by disrupting the activation and function of CAFs; and the alteration of ECM organization by targeting key molecules and pathways responsible for collagen fibrillogenesis and fiber arrangement within the ECM. In this section, we examine the use of biomechanical approaches to modulate the tumor ECM, thereby enhancing the intratumoral penetration of OVs.

### 3.2 Degradation of ECM components

#### 3.2.1 Degradation of tumor ECM collagen

Collagen is the most abundant protein in the tumor ECM and plays a pivotal role in determining its physical properties, such as density and stiffness (61). Additionally, collagen density is a critical factor influencing the intratumoral penetration of OVs (62). Consequently, therapeutic approaches that integrate OVI with collagen degradation hold promise for enhancing the intratumoral OV spread and oncolysis. Matrix metalloprotease (MMP), a diverse family of proteolytic enzymes is capable of degrading collagen, fibronectin, laminin, and PGs (63). To leverage MMPs for improving OV penetration, strategies include pre-treatment or co-administration with purified MMPs or engineering OVs to express MMP transgenes. Another approach is to modulate signaling pathways that regulate MMP expression in TME to boost intratumoral MMP secretion (**Table 1**). Among collagen degradation strategies, pre-treatment or co-administration of an OV with an MMP is the most direct method. For example, co-injection of oncolytic HSV-1 with bacterial collagenase has been demonstrated to enhance intratumoral distribution of HSV-1 in Mu89 melanoma tumors, leading to more potent and sustained tumor regression compared to HSV-1 treatment alone (54). However, a significant limitation of this approach is the transient activity of the injected enzyme, which can result in insufficient long-term enhancement of penetration.

**Table 1:** Pre-clinical studies reporting enhancement of intratumoral OV distribution by targeted degradation of ECM components.

Targeted Component	Therapeutic Strategy	OV Strain	Tumor Model	Effects on OV Penetration and Efficacy	Ref
Type IV collagen	Engineered MMP-9 expression by SK-N-AS neuroblastoma cells	GFP expressing HSV-1 (JD0G)	Spheroid and xenograft models of neuroblastoma	Diffuse JD0G distribution in both spheroid and xenograft SK-N-AS/MMP9 tumor masses.	(64)
	Engineered MMP-9 expression by OV	GFP-expressing HSV (KMMP9)	Spheroid and xenograft models of GBM	Widespread distribution of KMMP9 in spheroids and significant prolongation of animal survival.	(65)
	Induction of MMP expression by VEGF-A blockade followed by intratumoral injection of OV	Conditionally replicating adenovirus (CRAd-S-pk7)	Xenograft model of GBM	Anti-VEGF-A pre-treatment led to a greater than 2-fold increase in distribution and a 3-fold increase in replication of CRAd-S-pk7 associated with significantly increased tumor doubling time compared to control treatments.	(66)
Type I collagen	Engineered MMP-8 expression by OV	Non-replicating MMP-8 expressing adenovirus (AdMMP8) combined with wild-type adenovirus (Adwt300)	Xenograft tumor models of lung and pancreatic cancers	Diffuse distribution of Adwt300 after combined treatment resulted in longer survival in animals treated by the combination of the two viruses compared to those treated with the wild-type virus alone.	(67)
Multiple collagen types	Intratumoral co-injection of bacterial collagenase and replicating OV	Conditionally replicating HSV-1 (MGH2)	Xenograft model of melanoma	Combined treatment increased the area of MGH2 distribution more than 3-fold compared to MGH2 alone and was associated with more potent inhibition of tumor growth compared to either treatment administered alone.	(54)
Hyaluronic acid (HA)	Combination therapy of hyaluronidase (PH20)-expressing OV and PD-1 blockade	PH20-expressing oncolytic adenovirus (ICOVIR17)	Orthotopic GBM xenograft	ICOVIR17 treatment increased the infiltration of CD8+ T cells, promoted M1-polarization of TAMs, and induced the expression of PD-L1 in GBM cells. Moreover, ICOVIR17 showed synergistic effects with PD-1 blockade, resulting in increased survival rates in mice-bearing tumors	(68)
	Intravenous and intratumoral injection of hyaluronidase-expressing OV	Oncolytic adenovirus expressing hyaluronidase and RGDK motif (VCN-01)	Xenografts models of human pancreatic tumor and melanoma	VCN-01 treatment resulted in a dramatic decrease in the intratumoral hyaluronic acid content which was associated with disruption of stromal collagen organization. VCN-01 induced potent inhibition of tumor growth and resulted in significant improvement in animal survival across all tested tumor models	(69)
	Intratumoral injection of PH20 expressing OV	Oncolytic pseudorabies viruses (PRV) carrying PH20, IFN-γ and IL-18 (rPRV-IL-18-γ-PH20)	Pan02 murine pancreatic adenocarcinoma	rPRV-IL-18-γ-PH20 had widespread tumor cell necrosis and showed the most potent tumor growth inhibition compared to the other two OVs	(70)
	Intratumoral injection of PH20 expressing OV	Engineered vaccinia virus (KLS3020) expressing PH20, IL-12 and sPD1-Fc	CT26.WT and B16F10 syngeneic tumor models	Improved OV penetration and infiltration of CD4+ and CD8+ T cells in tumors which led to enhanced oncolysis and stimulation of robust systemic anticancer immunity	(71)
Chondroitin sulfate proteoglycans (CSPG)	Intratumoral injection of chondroitinase ABC-expressing OV	Oncolytic HSV-1–expressing bacterial Chase-ABC driven by the HSV-1 IE4/5 promoter	Spheroid and xenograft models of human glioma	Chase-ABC showed significant enhancement of OV spreading in glioma spheroids and *in vivo* studies showed a significant increase in viral titers, inhibition of tumor growth, and prolonged animal survival compared to animals treated with the parental rHscQ virus	(72)
Heparan sulfate proteoglycans (HSPG)	Dual virotherapy approach involving a conditionally replicating OV and a non-replicating heparanase-expressing virus	Conditionally replicating adenovirus (OBP-301) and non-replicating, heparanase-expressing adenovirus (Ad-S/hep)	Spheroid and orthotopic xenograft models of human mesothelioma	Ad-S/hep enhanced the penetration and transduction efficiency of OBP-301 which resulted in the enhancement of anti-tumor effects of OBP-301 in the orthotopic human pleural mesothelioma model	(73)

An alternative approach involves engineering either OVs or tumor cells to produce MMPs. For example, AdMMP8 is a replication-deficient adenoviral vector that encodes MMP-8, which targets type-I collagen for degradation (67). When co-administered with a wild-type (WT) adenovirus, the replication-incompetent AdMMP8 enhances the intratumoral penetration and distribution of the WT virus in lung and pancreatic cancer xenograft models (67). Similarly, a neuronal miRNA-sensitive, EGFR-targeted oncolytic herpes simplex virus (oHSV) equipped with an MMP-9 transgene demonstrated improved intratumoral spread, oncolysis, and survival rates in a glioblastoma xenograft model (65). Additionally, engineering tumor cells to express MMP-9, which degrades type IV collagen, has been shown to enhance the spread of oHSV in both spheroid and xenograft models of glioblastoma (64).

Alternatively, the intratumoral secretion of MMPs can be augmented by targeting the signaling pathways that govern MMP secretion. For example, anti-angiogenic therapies, such as anti-vascular endothelial growth factor A (VEGF-A) antibodies can induce the expression of MMPs (74). Pre-treatment with anti-VEGF-A antibodies resulted in an upregulation of MMP-2 expression in human U251 glioblastoma multiforme (GBM) xenografts and significantly enhanced the oncolytic effects of a subsequently administered conditionally replicating adenovirus (CRAd-S-pk7) (66). Similarly, signaling proteins, including hormones that modulate a spectrum of signaling pathways involved in fibrosis, have been utilized to diminish tumor desmoplasia and improve intratumoral OV dissemination. Relaxin, a peptide hormone, reduces fibrosis by inhibiting fibroblast proliferation, stimulating MMP expression, and repressing tissue inhibitor of metalloproteinases (TIMP) expression (75). In pancreatic cancer spheroid and xenograft models, an oncolytic adenovirus vector expressing relaxin (YDC002) decreased the expression of collagen, fibronectin, and elastin, while augmenting the cytotoxic effects of gemcitabine (76). Another relaxin-expressing oncolytic adenovirus (oAd/IL12/GM-RLX), armed with interleukin 12 (IL-12) and granulocyte-macrophage colony stimulating factor (GM-CSF), demonstrated the capacity to promote ECM degradation, facilitating the infiltration of activated and exhausted tumor antigen-reactive CD8+ T cells and enhancing the efficacy of ICB in refractory tumors (77).

#### 3.2.2 Degradation of hyaluronic acid

Hyaluronic acid is the simplest and most abundant GAG in the extracellular matrix, composed of a repeating disaccharide unit consisting of glucuronic acid and N-acetylglucosamine. HA significantly contributes to matrix organization by influencing the deposition of collagen and fibronectin fibers, and by modulating the activity of fibroblasts through the regulation of TGF-β signaling (78). Within the tumor ECM, HA is synthesized by various cell types, including tumor cells, stromal cells, and macrophages. Its accumulation is linked to an elevation in ECM stiffness and interstitial fluid pressure (79). In murine xenograft models of pancreatic cancer, HA accumulation has been associated with increased IFP, collagen content, vessel collapse, hypoxia, and metastasis, which were all attenuated following hyaluronidase (PH20) treatment (80).

Pre-clinical and early-phase clinical trial data offer promising insights into the efficacy of combining OVI with HA degradation. For example, VCN-01, an oncolytic adenovirus expressing PH20 and replicating selectively in cells with a defective retinoblastoma (pRb) pathway (69) has demonstrated favorable biodistribution and safety profiles in pre-clinical studies across various mouse and Syrian hamster models of melanoma, glioma, pediatric bone cancer, pancreatic cancer, and primitive neuroectodermal tumors (PNETs) (69, 81–83). Additionally, phase-1 clinical trials with metastatic and treatment-naïve solid tumor patients have yielded encouraging results. Treatment with intravenous VCN-01, either concurrently or neo-adjuvantly with chemotherapy (nab-paclitaxel and gemcitabine), has shown good virus biodistribution, a favorable safety profile, and clinical benefits in both concurrent and neo-adjuvant settings (84). Another PH20-expressing OV is rPRV-IL-18-γ-PH20, which is a pseudorabies virus expressing three transgenes including interleukin 18 (IL-18), interferon-gamma (IFN-γ), and PH20, and it has shown improved efficacy in preclinical experiments in cultured cells and mouse models of pancreatic cancer (Pan02) compared to control viruses rPRV-IL-18-γ and rPRV-PH20 (70). Likewise, KLS-3020, which is a recombinant oncolytic vaccinia virus containing three therapeutic transgenes including PH20, IL-12, and soluble programmed death protein 1 Fc (sPD1-Fc) has demonstrated enhanced OV penetration and immune cell infiltration in CT26.WT and B16F10 tumor models (71). Evaluation of viruses expressing single transgenes revealed the underlying mechanisms whereby PH20 promotes intratumoral virus spread and immune cell infiltration, IL-12 promotes activation of tumor-infiltrating T cells, while sPD1-Fc reduces intratumoral exhausted T cells. The ability of PH20-expressing OVs to promote immune cell infiltration can be harnessed to turn cold tumors hot and enhance the efficacy of ICIs. For instance, a PH20-expressing adenovirus, ICOVIR17 demonstrated synergistic effects with PD-L1/PD-1 blockade in an orthotopic murine model of GBM by suppressing HA-mediated inhibition of the nuclear factor kappa B (NF-Kb) pathway in macrophages, leading to their activation (68). The combination therapy increased T cell and macrophage infiltration in the tumors and enhanced animal survival compared to the control virus ICOVIR15, which lacks the hyaluronidase transgene. Several challenges may hinder the clinical translation of this strategy. First, intratumoral virus injection which was used in animal studies may not be ideal for patients with metastatic tumors due to the presence of multiple lesions. Therefore, obstacles to intravenous administration such as immune clearance of the viruses and poor tumor tropism will need to be addressed to allow intravenous administration. Second, there is a possibility of tumor cells developing resistance to virus infection, and therefore strategies to overcome this resistance are required in addition to overcoming ECM barriers.

#### 3.2.3 Degradation of proteoglycans

ECM proteoglycans play a pivotal role in collagen fibrillogenesis and organization, thereby influencing the mechanical properties of the matrix, including density, stiffness, and IFP (85, 86). These properties make them attractive targets for enhancing oncolytic virus penetration within desmoplastic tumors. Pre-clinical studies have indeed yielded encouraging results with proteoglycan targeting strategies. Chondroitin sulfate proteoglycans (CSPGs) are the most abundant PGs in the normal central nervous system (CNS) matrix and are often overexpressed in glioblastoma, where they are associated with increased ECM density (87). CSPGs are degraded by chondroitinase ABC (Chase-ABC), a bacterial polysaccharidase that specifically degrades the GAG chains of CSPGs (88). An oncolytic herpes simplex virus (OV-Chase) expressing Chase-ABC has demonstrated improved penetration within glioma models in both spheroid and xenograft settings (72). Treatment with OV-Chase resulted in significantly higher virus titers, tumor growth inhibition, and increased survival rates in treated animals compared to the control virus that lacked Chase-ABC (72). Efforts to enhance the stability and activity of Chase-ABC in mammalian tissues have led to the development of a mutant enzyme, ChaseM (89). Treatment with an oncolytic virus expressing ChaseM (OV-ChaseM) inhibited neurosphere formation *in vitro* and significantly improved median survival in glioma xenografts (90). The clinical efficacy of Chase-expressing oncolytic viruses can be further enhanced by dual intratumoral injection at the core and the periphery of tumor satellites (91).

Heparan sulfate proteoglycans (HSPGs) represent another class of proteoglycans that can significantly influence the physical properties of ECM. They can be targeted to normalize the dense tumor ECM and thereby enhance the penetrating ability of OVs. Heparanase is an endoglycosidase capable of breaking down heparan sulfate, a major component of the peritumoral ECM. An oncolytic adenovirus expressing heparanase (Ad-S/hep) has shown potent enhancement of the penetrating ability of a telomerase-targeted conditionally replicating oncolytic adenovirus (OBP-301) in human mesothelioma tumor spheroids (73). In an orthotopic xenograft model of human malignant pleural mesothelioma, concurrent intratumoral administration of OBP-301 and Ad-S/hep resulted in enhanced antitumor effects, as evidenced by a significant reduction in tumor weight and an extension in animal survival, compared to treatment with OBP-301 alone (73).

### 3.3 Inhibition of ECM synthesis

#### 3.3.1 Using engineered OVs to target myofibroblast CAFs

Apart from the degradation of an already deposited tumor ECM collagen, the tumor secretion, fibrillogenesis, and stiffening of the ECM can be controlled by inhibiting molecular pathways that govern collagen synthesis and organization. This can be achieved by targeting CAFs, either through the inhibition of their activation or the signaling pathways that control collagen expression (92) (**Table 2**). CAFs are a heterogeneous group of cells with distinct phenotypic and functional features that can be classified into several subtypes: myCAFs, iCAFs, rCAFs, and apCAF (92, 97, 98). The myCAF subtype is the primary contributor to ECM remodeling and is responsible for the secretion of various ECM proteins, including collagen, elastin, and fibronectin, as well as providing stromal contractility (99). A variety of signals, such as TGF-β, OX40L, PD1, prostaglandin E2 (PGE2), and B7-H3, mediate the cross-talk between CAFs and tumor-infiltrating immune cells, which is crucial for the maintenance of the CAF phenotype and the transition among the different CAF subtypes (92, 100).

**Table 2:** Preclinical studies reporting enhancement of intratumoral OV distribution by inhibition of ECM synthesis.

Therapeutic Strategy	OV Strain	Tumor Model	Effects of OV Penetration and Efficacy	Ref
Engineered expression of OX40L by OV	Herpes simplex virus-1 expressing murine OX40L (OV-mOX40L)	Syngeneic mouse model of pancreatic cancer	Treatment with OV-mOX40L reduced tumor growth, increased iCAF levels, decreased myCAF levels, reinvigorated intratumoral immune cells, activated CD4+ and CD8+ T cells, and reduced Tregs.	(93)
Combination therapy of relaxin-expressing OV and gemcitabine	Relaxin-expressing adenovirus (YDC002)	Spheroid and xenograft models of pancreatic cancer.	Expressions of collagen types I and III, fibronectin, and elastin were significantly inhibited in combined treatment compared to gemcitabine alone or control. In all models, YDC002 significantly inhibited tumor growth compared to control or gemcitabine alone and combined treatment showed synergistic effects.	(76)
Combination therapy of relaxin-expressing OV and ICB	Oncolytic adenovirus expressing relaxin, IL-12 and GM-CSF (oAd/IL12/GM-RLX)	Xenograft model of human gastric tumor and syngeneic hamster model of pancreatic tumor	oAd/IL12/GM-RLX showed enhanced ECM degradation, intratumoral penetration, and increased infiltration of CD4+ T cells and IFN-γ secretion. Combination treatment of oAd/IL12/GM-RLX and PD-1 blockade showed more potent inhibition of tumor growth compared to either treatment alone.	(77)
2-week intraperitoneal injection of losartan followed by two intratumoral OV injections 24 hours apart	Conditionally replicating HSV-1 (MGH2)	Xenograft models of human soft-tissue sarcoma (HSTS26T) and human melanoma (Mu89)	Losartan treatment decreased collagen I expression through inhibition of TSP-1 expression. Losartan significantly increased the intratumoral distribution of MGH2 in both HSTS26T and Mu89 tumor models.	(94)
Blockade of IL-6R by scFv expressed by engineered OV	Engineered adenovirus (LOAd713) expressing TMZ-CD40L and an scFv against IL-6R	Syngeneic mouse model of melanoma	LOAd713 treatment decreased multiple profibrotic factors including LAP-TGF-β1, type I collagen, FGF5, HGF, and TWEAK. LOAd713 increased immune cell infiltration, inhibited tumor growth, and prolonged survival in syngeneic mouse models of melanoma.	(95)
Combination treatment of anti-fibrotic drug (halofuginone) and OV	GFP-expressing vesicular stomatitis virus (VSV-GFP)	Syngeneic mouse colon carcinoma and human pancreatic cancer xenograft	Halofuginone treatment disrupted the tumor stromal barrier, decreased type I collagen, and broadened the intratumoral distribution of VSV-GFP. Combined treatment promoted DC maturation and CD8+ T cell activation, decreased Tregs, and increased animal survival.	(96)

The OX40/OX40L signaling interaction between stromal cells and T cells fosters a pro-inflammatory microenvironment by providing co-stimulatory signals for T cell activation, proliferation, survival, and inhibiting regulatory T cell (Treg) differentiation (101). Under these pro-inflammatory conditions, myCAFs undergo a phenotypic transition to iCAFs, which exhibit reduced ECM protein secretory activity (102). Consequently, the activation of OX40/OX40L signaling leads to a reduction in tumor ECM density and stiffness, thereby enhancing the intratumoral penetration of OVs. For instance, treatment with an engineered OX40-L expressing oncolytic herpes simplex virus-1 (OV-mOX40L) in a KPC syngeneic model of pancreatic carcinoma effectively remodeled the desmoplastic tumor ECM by increasing the number of iCAFs and decreasing the number of myCAFs (93). An OV-mOX40L treatment also increases the infiltration of CD4+ T cells, decreases the tumor-infiltrating CD8+ T cell exhaustion, reduces FOXP3+ regulatory CD4+ T cells, and reprograms macrophages and neutrophils to a pro-inflammatory state (93). Similarly, an engineered oncolytic adenovirus (LOAd713) that expresses an IL6 receptor (IL6R)-targeted scFv and a CD40 ligand (TMZ-CD40L) for simultaneous IL6R signaling blockade, and CD40 activation demonstrates enhanced T-cell infiltration. Furthermore, it reduces the PD-L1 expression in a syngeneic murine model of melanoma (95). Mechanistically, LOAd713 treatment inhibited the expression of multiple pro-fibrotic proteins, including TGF-β, type I collagen, fibroblast growth factor 5 (FGF5), hepatocyte growth factor (HGF), and TNF-like weak inducer of apoptosis (TWEAK), in pancreatic stellate cells (95).

#### 3.3.2 Pharmacological inhibition of ECM synthesis

To overcome ECM barriers that impede the penetration of OVs into tumors, antifibrotic drugs can be employed to inhibit ECM synthesis. This approach can be implemented through pre-treatment with an antifibrotic drug followed by an OV, or by concurrent administration of an OV and an antifibrotic drug. Several medications with established antifibrotic properties, which are already approved for other indications, can be repurposed to augment OV penetration. For example, losartan, an angiotensin II receptor blocker, possesses potent anti-fibrotic effects by inhibiting TGF-β signaling (103). Losartan treatment has been shown to reduce collagen I synthesis by CAFs *in vitro* and to decrease intratumoral collagen content in multiple murine tumor models, including those of mammary (FVB-MMTV-PyVT), pancreatic (L3.6pl), fibrosarcoma (HSTS26T), and melanoma (Mu89) (94). When combined with HSV, losartan treatment enhanced the intratumoral spread and efficacy of HSV in Mu89 and HSTS26T tumor models (94).

Halofuginone, a coccidiostat commonly used in veterinary medicine is another pharmaceutical agent with potent inhibitory effects on type I collagen synthesis. Its inhibitory effects are mediated through the repression of collagen I gene expression and the inhibition of the TGF-β signaling pathway (104, 105). In murine xenograft models of colorectal cancer, halofuginone significantly reduced stromal collagen content, as well as alpha smooth muscle actin (α-SMA) and fibroblast activation protein (FAP) expression (96). The combined treatment of oncolytic Vesicular Stomatitis Virus (VSV) and halofuginone significantly enhanced VSV intratumoral distribution, CD8+ T cell infiltration, and was associated with an extension in animal survival (96).

### 3.4 Modulation of ECM organization

The ECM is a complex network composed of collagen, elastin, fibronectin, laminin, and other glycoproteins secreted by cells, which collectively form a 3D meshwork. The intricate assembly of this 3D meshwork is facilitated by interactions between fibril-forming collagens and other ECM components, including other collagen types, fibronectin, integrins, and proteoglycans (106, 107). Within the tumor ECM, collagen fibrils exhibit a distinct organization from their counterparts in normal tissues. For instance, in lung adenocarcinoma, collagen fibers are more aligned, elongated, and straightened compared to the matched samples from normal lung tissues (108). This specific pattern of collagen organization in tumors enhances tumor cell invasiveness by facilitating migration along the radial direction (109). Likewise, in gastric cancer, collagen fibers display increased thickness, straightness, density, and cross-linking when compared to paired normal gastric tissues (110).

The organization of tumor ECM collagen is governed by both mechanical forces exerted by tumor cells and secreted factors. Tumor cells apply solid stress to the surrounding ECM, which stretches collagen fibers, leading to the characteristic straightened organization (111). Similarly, tumor cell-secreted matricellular proteins, such as WNT1-inducible-signaling pathway protein 1 (WISP1), bind to type I collagen, facilitating its linearization (112). Other key regulators of matrix organization include fibril-associated collagens (types IX, XII, XIV, XVI, XIX, XX, XXI, XXII, and XXVI), matrix proteoglycans, and fibronectin (113, 114). Therefore, targeting these ECM components that regulate collagen organization represents an attractive strategy to surmount the barriers imposed by the ECM on OV penetration.

Decorin (DCN) is a PG with significant ECM-organizing capabilities, playing pivotal roles in collagen fibril organization and stromal fibrosis through the suppression of TGF-β signaling in CAFs (113, 115, 116). DCN is composed of chondroitin or dermatan sulfate GAGs attached to a core protein containing leucine-rich repeats (LRRs) (117). It is a member of the family of small leucin-rich proteoglycans (SLRPs), which also includes lumican, biglycan, and fibromodulin (118). Decorin regulates multiple aspects of tumor biology, including tumor cell proliferation, migration, and exhibiting both pro-inflammatory and anti-fibrotic effects (119, 120). Engineered OVs expressing DCN have demonstrated improved intratumoral penetration in pre-clinical studies. A DCN-expressing adenovirus (Ad-DE1B-DCNG) displayed enhanced intratumoral penetration compared to control viruses carrying mutant decorin genes, which either cannot bind to collagen I fibrils (Ad-DE1B-DCNQ) or have reduced collagen I binding affinity (Ad-DE1B-DCNK) (121). Similarly, a hypoxia-responsive DCN-expressing oncolytic adenovirus (oH(E)mT-DCN) demonstrated enhanced intratumoral spread compared to control viruses lacking DCN expression in orthotopic mouse and patient-derived spheroid models of pancreatic cancer (122). Treatment with oH(E)mT-DCN attenuated the expression of multiple ECM components, including collagen I/III, elastin, and fibronectin, which further enhanced intratumoral virus spreading.

Furthermore, the ECM normalization effects of decorin expression offer opportunities for combination with other immunotherapies. By breaking down ECM barriers, the enhanced infiltration of both endogenous and exogenous immune cells, such as CAR-T cells can be facilitated (123). For example, a combination of a decorin-expressing oncolytic adenovirus (OAV-Decorin) and CAR-T cells targeting carbonic anhydrase IX (CAIX-CAR-T) was effective in reprograming, improving virus and CAR-T cell penetration, and prolonging survival in treated mice (124).

## 4 Physico-chemical strategies for tumor ECM disruption

Physico-chemical strategies for ECM disruption utilize magnetic, sound, and light energies to physically or chemically disrupt the tumor’s ECM. This section summarizes the applications of these strategies to enhance the intratumoral penetration of OVs, as reported in pre-clinical and clinical studies.

### 4.1 ECM disruption using focused ultrasound

Ultrasound is a versatile medical tool with a diverse range of applications, including diagnostic imaging, targeted delivery of therapeutics, and ablative treatment for various conditions (125, 126). The integration of microbubble technologies has further expanded the clinical applications of ultrasound, enabling advancements in image contrast enhancement, *in-situ* manipulation for targeted delivery, penetration enhancement, and ablative treatment (127). As a method for targeted drug delivery, ultrasound provides the ability to control the spatiotemporal release of therapeutic agents, thereby enhancing their therapeutic efficacy (128). This is accomplished through the encapsulation of payloads into surface-targeted microbubbles, followed by their controlled release mediated by intratumoral exposure to focused ultrasound (129, 130).

Accumulating evidence supports the efficacy of focused ultrasound in facilitating targeted delivery and enhancing intratumoral penetration of oncolytic viruses. The integration of microbubble technology with ultrasound enables targeted delivery, mitigates immune clearance, and enhances intratumoral penetration of oncolytic viruses (131). Microbubbles can encapsulate the OV to avert immune system clearance, and controlled intratumoral release of viruses can be achieved by utilizing high-intensity focused ultrasound to destroy microbubbles (132). For instance, the use of ultrasound-mediated polymeric nanocup destruction resulted in a significant improvement in the intratumoral penetration of oncolytic vaccinia virus (VV) in xenograft models of liver and colon tumors (133). This enhancement was attributed to sustained inertial cavitation effects that propelled the intravenously co-administered VV by hundreds of microns, correlating with a corresponding increase in reporter gene expression and the number of recovered VV genomes (133). Similarly, intravenous co-injection of a luciferase-expressing adenovirus (AdEHE2F-Lu) and microbubbles followed by exposure to focused ultrasound significantly augmented the delivery and intratumoral distribution of the virus in a mouse model of breast cancer (134). Furthermore, ultrasound-triggered inertial cavitation of gas microbubbles enhanced the penetration of polymer-coated adenovirus up to 100 µM from the nearest blood vessel, leading to approximately a 30-fold enhancement of tumor cell infection and significant impairment of tumor growth, ultimately prolonging survival in treated mice (135).

**Figure 4:**
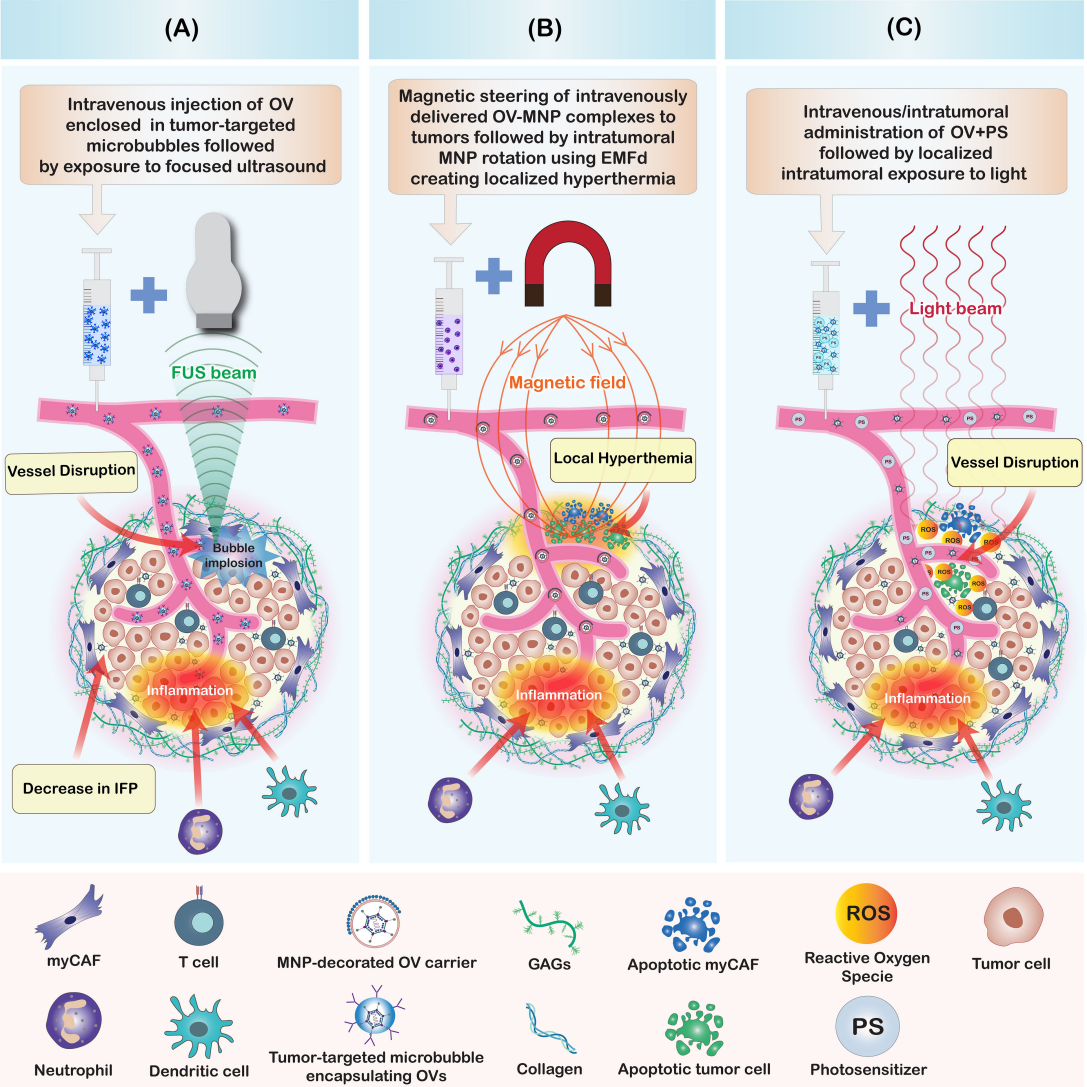
Summary of physico-chemical strategies to enhance oncolytic virus penetration in tumors. **(A)** Focused ultrasound in combination with microbubbles enhances OV penetration by creating shear stress and cavitation effects. This mechanism leads to a disruption of intratumoral blood vessels, induction of intratumoral inflammation, reduction of intratumoral interstitial fluid pressure (IFP), and a disruption of cell-cell contacts and tissue structure. Collectively, these alterations improve the penetration of intravenously injected OVs within the tumor. **(B)** Magnetic nanoparticle-decorated OV carriers enable directional steering to tumor sites. The intratumoral rotation of MNP-coated particles, induced by an externally applied magnetic field (EMF) generates localized hyperthermia within the tumor. This results in blood vessel disruption, tumor and stromal cell death, and the induction of stromal inflammation, all of which enhance the intratumoral penetration of OVs. **(C)** Photodynamic therapy (PDT) enhances the intratumoral penetration of OVs. Illumination of the tumor with light, following the intratumoral or intravenous co-administration of OVs combined with photosensitizers (PS) or photoactivatable OVs (PD-OVs), generates reactive oxygen species (ROS) in the tumor microenvironment. These ROS induce stromal and tumor cell death, blood vessel disruption, and inflammation, thereby improving the intratumoral penetration of both OVs and immune cells.

The enhancement of intratumoral OV penetration achieved through focused ultrasound is facilitated by several mechanisms (**Figure 4A**). One mechanism involves the creation of fluid or solid shear stress in the vicinity of oscillating microbubbles, which disrupts blood vessels and facilitates OV extravasation (136). Another mechanism is the generation of cavitation effects through ultrasound-targeted microbubble destruction (UTMD), which enhances intratumoral delivery and distribution of OVs by increasing tumor perfusion, extravasation, and matrix penetration (129, 137). Other mechanisms include the induction of intratumoral inflammation resulting from UTMD disruption of cell-cell adhesions and tissue structure (138, 139), and the reduction of intratumoral interstitial fluid pressure, which allows for greater penetration of OVs towards the central areas of the tumor parenchyma (140). These mechanisms collectively enable UTMD to effectively overcome physical and immunological barriers of TME to enhance the efficacy of immunotherapy in poorly responsive tumors (141, 142).

Possible limitations to microbubble-based strategies include the limited half-life of microbubbles in the circulation and tissue concerns of tissue injury due to excessive pressure (143). In addition, the possibility of damaging the encapsulated OVs which may reduce their oncolytic activities need careful consideration. This can be achieved by titrating the frequency of ultrasound by assaying the therapeutic activity of the payload is maintained under expected exposure conditions. For instance, by carefully regulating the level and duration of exposure, it is possible to achieve enhanced penetration of oncolytic adenovirus and vaccinia viruses, as well as other therapeutics, without compromising their therapeutic efficacy (144).

### 4.2 ECM disruption using magnetic nanoparticles

Magnetic nanoparticles (MNPs) are magnetic nanocarriers that are manipulatable via the application of a magnetic field. MNPs provide means to potentiate oncolytic virotherapy by facilitating directional delivery, bypassing immune clearance, disrupting the tumor ECM, and enhancing virus transduction into tumor cells (145–148). The destruction of tumors and their stromal components by MNPs can be accomplished through mechanical and thermal strategies (**Figure 4B**). Mechanically, an external magnetic field (EMF) is applied to induce MNP rotation, which results in damage to cancer and stromal cells within the TME (149, 150). Alternatively, the rotation of MNPs generates localized temperature increases (hyperthermia), leading to the death of cancer cells and cancer-associated stromal cells, thereby limiting tumor growth and reducing ECM stiffness (151, 152). The MNP-induced disruption of tumor stroma facilitates the penetration of therapeutics and the infiltration of tumor antigen-specific T cells, thereby sensitizing resistant tumor cells to immunotherapy and other conventional therapies (153).

Choi et al. (146) investigated a strategy to enhance the therapeutic efficacy of oncolytic adenoviruses (oAd) by conjugating viral particles with MNPs. They created MNP-OV complexes by combining a firefly luciferase-expressing, conditionally-replicating oncolytic adenovirus (HmT) with PEGylated and cross-linked iron oxide nanoparticles (PCION) to form HmT-PCION complexes. In MCF-7 xenograft models, magnetically-guided HmT-PCION complexes demonstrated enhanced therapeutic efficacy while significantly reducing the non-specific hepatic tropism of the virus (146). In another study, magnetic nanoparticles derived from a magnetotactic bacterial strain (*Magnetospirillum magneticum* AMB-1) were utilized to increase the penetration of oncolytic HSV-1 in pre-clinical mouse models of breast cancer (154). Here, magnetized OVs (MAG-OV) were created by electrostatically complexing bacterial-derived magnetosomes (AMB-1 MAG) with HSV1716-GFP, followed by magnetic targeting to the tumor after intravenous administration of the magnetized OVs. Magnetically targeted MAG-OV (MAG-OV+MT) exhibited enhanced *in-vivo* antitumor effects, including the inhibition of tumor growth, reduction of metastasis, and improvement of animal survival. Furthermore, MAG-OV+MT was able to induce immunogenic cell death characterized by the extracellular release of ATP and HMBG1, accompanied by an increase in tumor-infiltrating immune cells such as natural killer (NK) cells, macrophages, CD8+ T cells, and neutrophils, as well as a decrease in infiltrating B cells (154).

An alternative approach for utilizing MNPs to enhance intratumoral penetration of OVs involves the decoration of carrier cells with MNPs to control the steering of the magnetized complexes using an EMF (155–157). In a study employing this strategy, oncolytic adenovirus-infected 293T carrier cells were surface-modified by adding cyclic arginine-aspartic acid (cRGD) for bladder targeting and asymmetrically immobilizing Fe3O4 MNPs (155). These carrier cells were successfully steered by EMF to facilitate their intratumoral accumulation and retention. Similarly, magnetic steering of asymmetrically Fe3O4-coated, kidney-targeted Janus cell robots enabled their navigation through confined spaces and migration from the bladder to the kidneys (156).

These pre-clinical studies underscore the potential of MNPs to enhance the delivery, infection, penetration, and intratumoral retention of OVs, thereby bolstering their therapeutic efficacy. However, the clinical translation of this strategy may face several challenges including difficulty in removing the MNPs once administered in the body, biocompatibility issues, and concerns of toxicity and tissue damage (158). Further research in clinically relevant animal models is essential to facilitate the clinical translation of these strategies.

### 4.3 ECM disruption using photodynamic therapy

Photodynamic therapy (PDT) is a form of cancer treatment that harnesses light energy to activate and excite light-sensitive drugs known as photosensitizers. When exposed to a specific wavelength of light, these photosensitizers transition to an unstable excited state (159). The excited photosensitizers then transfer energy to triplet oxygen or other substrates, such as lipids, proteins, or nucleic acids, triggering a series of redox reactions that lead to the production of reactive oxygen species (ROS) that damage cells (160). The ROS-mediated cell damage leads to a range of death pathways and mechanisms that include apoptosis, autophagy, necrosis, necroptosis, paraptosis, mitotic catastrophe, pyroptosis, and parthanatos (161). A key advantage of PDT is its ability to target specific areas, enabling tumor ablation with minimal damage to healthy tissues (162). This is made possible by engineering photosensitizers to specifically target tumors and using precise application of light to target tumor tissues. PDT can be combined with other treatment modalities such as radiotherapy, chemotherapy, hyperthermia, cold plasma therapy, sonodynamic therapy, and immunotherapy (163).

The rationales for combining PDT and OVI include the possibility of engineering photodynamic OVs (PD-OVs) that express photosensitizer proteins to facilitate their tumor targeting, PDT-mediated disruption of tumor stroma including blood vessels allowing intratumoral accumulation of intravenously administered OVs (164), and use of CAF-targeted photosensitizers to ablate CAFs and inhibit ECM synthesis (165) (**Figure 4C**). In one study, the combination of PDT and OVI was investigated using intravenous injection of the 2-[1-hexyloxyethyl-]-2-divinyl pyropheophorbide-a (HPPH) photosensitizer and a green fluorescent protein (GFP)-expressing oncolytic vaccinia virus (OVV-GFP) (164). The aim of the combination therapy was to leverage the vascular-disrupting effects of PDT to augment the OVV accumulation in tumors. The results showed that PDT in combination with OVV-GFP led to tumor vascular disruption, which in turn enhanced intratumoral viral titers and exhibited the highest antitumor efficacy compared to the use of PDT or OVV-GFP alone (164).

Photodynamic-oncolytic viruses (PD-OVs) are created by integrating a gene encoding a photosensitizer protein, such as KillerRed, into pre-characterized OVs (166–170). KillerRed is a genetically encoded photosensitizer derived from a hydrozoan chromoprotein (anm2CP) (168). An example of a PD-OV is TelomeKiller, which is a KillerRed-expressing, telomerase-specific replication-competent oncolytic adenovirus engineered by inserting the KillerRed expression cassette into the E3 region of OBP-301 (Telomelysin) (170). TelomeKiller showed potent oncolytic activity following intratumoral injection into the HCT116 xenograft model of metastatic colorectal carcinoma, leading to the formation of large necrotic areas within the treated tumors (170). G47Δ-KR is another KillerRed-expressing OV engineered from oncolytic herpes simplex virus (oHSV) (166). In xenograft models of GBM and malignant meningioma (MM), intratumorally injected G47Δ-KR followed by laser irradiation showed more potent oncolytic activity compared to G47Δ-KR or laser irradiation alone. The combination treatment also led to intratumoral infiltration of immune cells, reflecting the PD-OV’s ability to breach physical and immunological barriers within TME. Another KillerRed-expressing PD-OV was generated by engineering mammalian orthoreovirus (MRV) to express membrane-targeting KillerRed (KRmem) (169). This PD-OV demonstrated enhanced cytotoxicity in gastric cancer cell lines compared to the wild-type MRV, although *in-vivo* experiments to evaluate its impact on stromal barriers were not performed.

The pre-clinical studies on the combination therapy of PDT and OVI provided promising evidence of treatment efficacy. The encouraging outcomes from these studies justify the pursuit of further investigation through translational research and clinical trials. However, it is crucial to acknowledge that the translation of these promising results into clinical applications will encounter significant challenges. They include the difficulties in delivering illumination to deeply seated tumors and the potential inactivation of OVs by PDT. Additionally, the hypoxic tumor microenvironment presents another obstacle that must be addressed to ensure the effectiveness of the combination therapy.

## 5 Effects of ECM-targeted OVs on TIME

In addition to enhancing the intratumoral penetration and distribution of OVs for an effective oncolysis, ECM-targeted OVs can reprogram TIME to facilitate anti-cancer immune reactions. An important characteristic of OVs is their ability to increase the infiltration of T-cells into tumors, effectively converting immunologically cold tumors into hot (171). This conversion is achieved through OV-mediated tumor cell lysis, which releases TAs and DAMPs. These are then taken up by intratumoral dendritic cells (DCs) for priming of T-cells in tumor-draining lymph nodes. The ability of ECM-targeted OVs to increase T-cell infiltration in cold tumors arises from two primary mechanisms: (i) the enhanced efficiency of tumor cell lysis, leading to the release of TAs and DAMPs, and (ii) the overcoming of barriers imposed by the ECM on T-cell trafficking and infiltration of T-cells into tumors (172).

Several oncolytic adenoviruses engineered to carry a therapeutic transgene for PH20 have shown a high efficacy in improving the infiltration of T cells into cold tumors (68, 71, 173). VCN-01 is an example of an ECM-targeted oncolytic adenovirus that carries a PH20 transgene for intratumoral degradation of hyaluronic acid. In a phase I clinical trial involving patients with advanced or metastatic pancreatic cancer, VCN-01 enhanced the infiltration of CD8+ T cells and the upregulation of indole 2,3-dioxygenase, resulting in clinical response rates of 40 to 45% (173). VCN-01 has been granted orphan drug designation for the treatment of retinoblastoma and PDAC (174). ICOVIR17 is another PH20-expressing oncolytic adenovirus that demonstrated improved tumoral infiltration of CD8+ T-cells in an animal model of glioblastoma (68). ICOVIR17 treatment also increased PD-L1 expression by tumor cells and enhanced the efficacy of PD-1 blockade. Owing to their large genome size and ability to carry multiple therapeutic transgenes (175), oncolytic adenoviruses have been engineered to express both PH20 and immunostimulatory molecules for modulating phenotypes of infiltrating T cells. For instance, intratumoral injection of an oncolytic adenovirus armed with therapeutic transgenes for PH20, IL-12, and sPD1-Fc (KLS3020) resulted in increased tumoral infiltration of CD4+ and CD8+ T cells, intratumoral populations of IFN-γ+ effector CD4+ and CD8+ T cells, and enhanced the Teff/Treg ratios of tumoral TILs in animal models of melanoma and colorectal carcinoma (71). These effects led to the activation of systemic anti-cancer immune reactions, which controlled tumor growth in non-injected lesions. Similar effects were observed with a recombinant pseudorabies virus carrying transgenes for PH20, IL-18, and IFN-γ expressing PRV (rPRV-IL-18-γ-PH20), further demonstrating the efficacy of ECM-targeting OVs in improving T cell infiltration of tumors and inducing robust systemic anti-cancer immune reactions (70).

ECM-targeting OVs that inhibit the synthesis of ECM components can also enhance the intratumoral infiltration of T cells. For instance, an oncolytic herpes simplex virus (HSV-1) expressing murine OX40L (OV-mOX40L) led to increases in tumoral CD4+ and CD8+ TILs in animal models of pancreatic ductal adenocarcinoma (93). The OV-mOX40L treatment also increased the expression of effector CD8+ T cells expression of IFNγ and GZMB, and decreased the expression of inhibitory receptors, such as PD-1 and LAG-3 (93). Similarly, an increase in intratumoral infiltration of CD8+ T-cells was observed with 0X40L expressing OV (LOAd713) in animal models of pancreatic cancer (95). The addition of immunomodulatory genes further enhances the phenotype of infiltrating T cells by increasing the number of effector TILs. For instance, an oncolytic adenovirus carrying a therapeutic gene for the anti-fibrotic hormone relaxin (RLX) and genes for immunomodulatory cytokines IL-12 and GM-CSF (oAd/IL12/GM-RLX) increased the intratumoral infiltration of activated CD4+, IFN-γ+ and CD8+, IFN-γ+ T cells in a Syrian hamster model of pancreatic cancer (77). Combination therapy of oAd/IL12/GM-RLX with αPD1 increased the proportions of intratumoral CD8+, IFN-γ+ and CD8+, PFRN+ T cells, leading to a durable suppression of tumor growth.

In addition to converting cold tumors to hot by increasing their T cell infiltration, ECM-targeted OVs contribute to a global improvement in TIME, addressing immunosuppression and enhancing anti-cancer immune responses. These effects include overcoming the exhaustion of TILs (93), reducing the presence of immunosuppressive cells such as Tregs (71, 93, 176), M2-like TAMs (68, 93, 176), and myeloid-derived suppressor cells (MDSCs) (95, 176), as well as DC maturation and antigen presentation (176). These collective effects position ECM-targeted OVs as an effective tool to augment anti-cancer immune reactions, thereby enhancing the efficacy of immunotherapy in refractory tumors.

Due to their dual capabilities of inducing ICD and enhancing OV penetration, physico-chemical strategies for tumor ECM disruption have the potential to profoundly modulate TIME and enhance anti-cancer immune reactions. For instance, in mouse models of glioblastoma and malignant meningioma, PDT using a KillerRed-expressing engineered oncolytic HSV increased the infiltration of a diverse range of immune cells, including lymphocytes, NK cells, monocytes, macrophages, and neutrophils (166). Similarly, magnetically-steered MNP-encapsulated OVs triggered an increase in tumoral infiltration of activated immune cells, such as neutrophils, cytotoxic T-cells, NK cells, and macrophages, as well as a decrease in tumoral infiltration of Tregs and B cells (154).

## 6 Clinical trials of ECM-targeting OVs

Several ECM-targeting strategies have shown promising outcomes in pre-clinical studies, as outlined in **Tables 1**, **2**. However, the majority of these strategies have not yet been evaluated in clinical trials. Clinicaltrials.gov lists twelve registered clinical trials involving three ECM-targeted OVs: VCN-01, LOAd703, and DNX-2440. These trials are predominantly early-phase (phase I or phase I/II) and are focused on assessing the safety and optimal dosage of the OVs when used as a single agent or in combination with other treatments, including ICB, CAR-T therapy, and cytotoxic chemotherapy (**Table 3**). A significant proportion of these trials employ combination therapy, with only three using OVI monotherapy. Four trials have reached completion, three are still actively recruiting participants, and two have been terminated due to resource limitations at the treating centers (NCT03555149) and stock issues (NCT03714334).

**Table 3:** Clinical trials of tumor ECM targeted OVs.

Trial Number	OV	Combination Therapy	Cancer Type	Phase	Status
NCT03284268	VCN-01	-	Recurrent Retinoblastoma	I	Recruiting
NCT05057715	VCN-01	CAR-T Cells	Pancreatic Cancer, Serous Ovarian Cancer	I	Recruiting
NCT02045589	VCN-01	Gemcitabine, Paclitaxel	Advanced Pancreatic Adenocarcinoma	I	Completed
NCT02045602	VCN-01	Gemcitabine, Paclitaxel	Locally Advanced and Metastatic Solid Tumors	I	Completed
NCT05673811	VCN-01	Paclitaxel, Gemcitabine	Metastatic Pancreatic Adenocarcinoma	IIb	Recruiting
NCT03799744	VCN-01	Durvalumab	Metastasis/Recurrent Squamous Cell Carcinomas of Head and Neck	I	Active, not recruiting
NCT03225989	LOAd703	Gemcitabine, SoC chemotherapy	Pancreatic, biliary, colorectal, and ovarian cancers	I/II	Active, not recruiting
NCT04123470	LOAd703	Atezolizumab	Malignant melanoma	I/II	Completed
NCT02705196	LOAd703	Gemcitabine, nab-paclitaxel	Pancreatic cancer	I/II	Completed
NCT03555149	LOAd703	Regorafenib, atezolizumab, Imprime PGG, bevacizumab, isatuximab, selicrelumab, idasanutlin, AB928	Colorectal cancer	Ib/II	Terminated
NCT04714983	DNX-2440	–	Colorectal cancer with resectable liver metastasis	I	Unknown
NCT03714334	DNX-2440	-	Recurrent glioblastoma	I	Terminated

Two completed phase I trials evaluated the safety and preliminary efficacy of VCN-01in patients with pancreatic and other advanced solid malignancies. In the first trial, VCN-01 was administered intratumorally to pancreatic cancer patients in combination with either gemcitabine or gemcitabine plus nab-paclitaxel. The treatment was well-tolerated and led to disease stabilization (177). The dose-limiting side effects included asthenia, grade three serum transaminasemia, and a fatal pancreatic fistula in one patient. The efficacy analysis revealed that all treated lesions remained stable, with new lesions appearing in five patients after four months, in one patient after eight months, and in another patient after 31 months. In the second trial, VCN-01 was administered as a single intravenous infusion of VCN-01 to 16 patients with locally advanced or metastatic solid tumors, or as part of two combination schedules with gemcitabine and nab-paclitaxel to 26 patients with pancreatic cancer (84). The treatment demonstrated an acceptable safety profile with an encouraging efficacy. The dose-limiting side effects included one patient with grade 4 raised aspartate aminotransferase (AST), one patient with grade 4 febrile neutropenia, and one patient with fatal thrombocytopenia and enterocolitis. In patients with metastatic PDAC, the combination therapy of VCN-01 and nab-paclitaxel and gemcitabine resulted in an overall treatment response rate of 50%, with 36% of patients showing stable disease for more than 12 months. *Post-hoc* analyses showed a median progression-free survival of 7.2 months and a median overall survival of 13.4 months. These outcomes are superior to those reported with combination therapy of oncolytic reovirus (Pelareorep) in combination with gemcitabine which resulted in an objective response of 3% and a median overall survival rate of 10.2 months (178).

Another two completed trials are phase I/II studies assessing the safety and efficacy of a TMZ-CD40L-expressing oncolytic adenovirus (LOAd703) in pancreatic cancer and melanoma patients. In one of these trials (179), LOAd703 was administered intratumorally in combination with standard nab-paclitaxel/gemcitabine (nPG) chemotherapy to patients with advanced pancreatic cancer. The combination therapy was found to be tolerable and feasible with observed side effects attributed to LOAd703 being low-grade and short-lived, except for one patient with grade 3 transaminasemia. LOAd703 treatment resulted in an overall response rate (ORR) and disease control rate (DCR) of 44% and 94% respectively, with corresponding immune activation. A follow-up study by the same research group is focused on evaluating a combination therapy of intratumoral LOAd703 and intravenous anti-PD-L1 monoclonal antibody atezolizumab in melanoma patients. The results of this study have not yet been published. The results of these completed phase I/II trials indicate the feasibility and safety of ECM-targeted OVs and suggest potential clinical benefits in the treatment of advanced and treatment-resistant solid tumors such as pancreatic cancer and melanoma.

## 7 Current limitations of ECM-targeted OVs

Despite promising results in pre-clinical studies of ECM-targeted OVs, several limitations may hinder their clinical translation. In most animal studies, intratumoral injection is the most commonly used delivery method to avoid recognition and clearance of the viruses by the host immune system. However, intratumoral administration is not feasible in patients with metastatic tumors due to the presence of multiple lesions, some of which may not be detectable. This assertion is supported by the results of a phase I trial in metastatic PDAC which showed disease progression in non-injected, distant metastatic sites (177). To overcome this limitation, OVs can be encapsulated using suitable tumor-tropic delivery vehicles such as extracellular vesicles, cells, or polymers (180–182). Furthermore, most of the engineered ECM-targeted OVs are directed to a single ECM component, and thus can only be effective in tumors with dysregulation of that particular component. For instance, collagenase-expressing OVs such as KMMP9 and JD0G will only be effective in tumors with aberrant collagen expression and similarly, hyaluronidase-expressing OVs such as VCN-01 and ICOVIR17 will only be effective in tumors with overexpression of hyaluronic acid. However, the composition of the tumor ECM is highly heterogeneous resulting in significant differences in relative quantities of different ECM components within tumors of similar histological types (183, 184). This necessitates the pre-treatment proteomic characterization of the tumor ECM to determine dysregulated ECM components for personalized treatment approaches. Alternatively, ECM-targeted OVs can be engineered to target multiple ECM components that are ubiquitously overexpressed in tumors. Another important consideration for ECM-targeted virotherapy is the possibility of enhancing tumor cell dissemination by breaking the ECM barriers. This is due to the role played by the tumor ECM in tumor cell confinement, proliferation and migration (185). The release of the ECM barrier may thus increase the number of circulating tumor cells to enhance metastatic dissemination of the treated tumors. Moreover, the physicochemical strategies of ECM disruption face several drawbacks including possibility of normal tissue injury due to inability to specifically target tumor cells. Also, the rapid and poorly controlled disruption of the ECM barriers by focused ultrasound, MNPs or PDT may enhance the dissemination of previously confined tumor cells.

Addressing these limitations will enhance the clinical translation of ECM-targeted virotherapy to enhance the penetration and distribution of OVs in tumors. The resulting improvement of tumor cell lysis and release of DAMPs will enhance immune cell infiltration into tumors, converting cold tumors into hot. This will open new possibilities of combination immunotherapies of OVs and ICIs, cancer vaccines or cellular therapies.

## 8 Future directions of ECM-targeted OVs

Due to vast heterogeneity in the composition of tumor ECM both between and within tumor subtypes, personalized treatment approaches will enhance the future utilization of ECM-targeted OVs. Personalized treatment approaches are made possible due to the rich arsenal of ECM-targeted OVs that are directed to virtually all ECM components including collagen, hyaluronic acid, and proteoglycans. Central to personalized approach in ECM-targeted OVs is the profiling of individual patient’s ECM components to inform personalized selection of ECM-targeted OVs that patient-specific dysregulated ECM components. The characterization of individual patient’s ECM proteome can be achieved by using several assays such as ELISA, immunohistochemistry (IHC) or mass spectrometry-based approaches (186). Of these assays, IHC is the most attractive option due to its ability to provide both quantitative and spatial information about the abundance and localization of ECM components in the tumor stroma (187). Furthermore, IHC is a routine test in majority of oncology hospitals and thus does not require significant capital investments in terms of human resource and equipment. The implementation of personalized approaches will ensure the right ECM-targeted OV for the right patient, which can lead to substantial improvements in the outcomes of treatment.

## 9 Conclusion

Oncolytic virus immunotherapy holds great promise in the fight against cancer, offering specificity and the ability to stimulate long-term anti-cancer immune responses. However, several barriers hinder the effectiveness of OVs in infecting, replicating, and lysing tumor cells, including dense and stiff tumor ECM, abnormal vasculature, and elevated intratumoral IFP. To address these challenges, various strategies have been developed, including targeted degradation of ECM components, inhibition of collagen synthesis, modulation of ECM organization, and the use of mechanical disruption techniques. Despite the lag in clinical translation, Phase I clinical trials of VCN-01, LOAd703, and DNX-2440 have demonstrated an acceptable safety profile and early evidence of clinical efficacy in a range of solid tumors. It is important to note that a re-normalization of the ECM by matrix-targeting OVs not only can enhance the intratumoral distribution of other therapeutics, such as chemotherapy drugs, monoclonal antibodies, and CAR T cells, but it can also convert cold tumors into hot tumors, thereby enhancing the efficacy of immunotherapies. Pre-clinical studies have demonstrated the benefits of combination treatments involving ECM-normalizing OVs with immune checkpoint inhibitors, CAR-T cells, TIL therapy, and cancer vaccines. Lastly, physicochemical strategies for disrupting the tumor stroma, such as UTMD, magnetic steering of MNP-decorated OVs, and photodynamic therapy offer alternative approaches to improving intratumoral OV distribution. These approaches have shown promising results in pre-clinical studies and have the potential to reduce treatment-related adverse events. The realization of the potential of OVs as a tool for transforming immunologically cold tumors into hot tumors will enhance the efficacy of immunotherapy and broaden the scope of patients who can benefit from immunotherapy.
